# The tendency of segmental distribution of hepatic metastasis according to couinaud classification: a comparison of portal versus systemic route of metastatis due to primary colorectal and breast tumors

**DOI:** 10.1097/MS9.0000000000001241

**Published:** 2023-09-04

**Authors:** Dawar Khan, Ayimen Khalid Khan, Sarim Dawar Khan, Muhammad Aman, Ahsun Amin, Maria Waseem, Usha Kumari, Faheemullah Khan, Alina Pervez, Aarash Khan

**Affiliations:** Departments ofa Radiology; b Medicine, Aga Khan University Hospital; c Aga Khan University Hospital; d Dow University of Health Sciences, Karachi, Pakistan; e Herat University, Herat, Afghanistan

**Keywords:** breast tumour, liver metastasis, portal venous route, primary colorectal cancer, systemic venous route

## Abstract

**Objective:**

The liver is the commonest site of metastatic disease for patients with colorectal cancer (CRC), with at least 25% of patients developing liver metastasis (LM) during their illness. About 50% of patients diagnosed with metastatic breast cancer develop LM, and 5–12% of these patients develop LM as the main site of breast cancer recurrence. This study aims to determine the frequency of segmental distribution of LM seeding from portal versus systemic routes of dissemination due to primary CRC and breast carcinoma, respectively.

**Material and methods:**

This retrospective study was conducted in a tertiary care teaching hospital in Pakistan. Ethical approval was taken from the institutional review board. A total of 587 patients were included in the study with 297 CRC patients with LM and 300 breast carcinoma patients with LM. Segment I involvement was excluded from the calculation because of the dual blood supply. Patients’ detailed demographics and other information were collected on a predesigned proforma. The authors evaluated axial and multiplanar reformatted computed tomography images for LM and CRC metastasis. Data analysis was done using SPSS version 25. *P* value less than or equal to 0.05 was considered statistically significant.

**Results:**

A study population of 587 patients was employed that comprised 287 CRC and 300 breast carcinoma patients. There were 179 (30.5%) male and 408 (69.5%) female patients. The mean age of patients was 54.9±13.3. The study revealed that 204 (34.8%) CRC patients showed right lobe (V, VI, VII, VIII) and 83 (14.1%) CRC patients showed left lobe involvement of metastasis while 192 (32.7%) breast carcinoma patients showed right lobe involvement and 108 (18.4%) breast carcinoma patients showed left lobe involvement in metastasis (*P*=0.02). We also found 40 (6.8%) colorectal and 55 (9.4%) breast carcinoma patients showed left lateral segment (II, III) involvement. Medial segment involvement (IV) was seen in 43 (7.3%) CRC patients and 53 (9%) breast carcinoma patients (*P*=0.03).

**Conclusion:**

The right hepatic lobe is the predominant site of metastasis independent of the portal or systemic route of dissemination in primary carcinoma. Moreover, in left lobe metastasis medial segment (IV) is more affected in CRC while the lateral segment (II, III) is more affected in breast carcinoma.

## Introduction

Highlights

- Liver is the main site of metastasis due to portal circulation which is source of two-thirds of liver blood.
- This study highlights frequency of hepatic metastasis of colorectal cancer and breast carcinoma.
- It is concluded that segments I and II lesions were more common in women with breast cancer, while segment VII lesions were more frequent in the colorectal cancer patients.

Colorectal carcinoma (CRC) is the leading cause of mortality and the third most common cause of cancer worldwide^1^. CRC is associated with two disease spectra, depending on the location and laterality of the primary tumour^2^. It can be characterized based on survival rate, progression of the disease, and metastatic spread^3^. According to the localization of the tumour, a difference in genetic and epigenetic features is also found. However, the difference in the behaviour of the tumour might be attributed to the embryological origin of the tissue involved (mid and hindgut)^4^.

Breast cancer is the most common cancer diagnosed in women, accounting for more than 1 in 10 new cancer diagnoses each year. It is the second most common cause of death from cancer among women worldwide^5^. Despite being one of the leading causes of mortality in post-menopausal women, most cases are still diagnosed in advanced stages due to negligence towards screening^6^. Identification of factors associated with an increased incidence of breast cancer development is imperative for better health screening in women^7^.

CRC continues to be the third leading cause of cancer-related mortality with the lifetime risk of developing CRC of about 1 in 23 for men and 1 in 26 for women, predicting about 52 550 deaths during 2023 in the United States alone^8^. Literature reported that 19% of CRC patients present usually with distant metastasis at the time of diagnosis^9^. Current studies suggest that about 35–55% of patients diagnosed with CRC will develop hepatic metastasis with long-term survival depending on surgical resection^10^. Literature reported a 90% survival rate at the early stage while this rate decreases by 10% with advanced-stage distant metastasis. Due to portal venous circulation, the liver is a common site of metastasis. An estimated two-thirds of liver blood drives from the portal circulation. The inferior mesenteric vein, splenic vein, and superior mesenteric vein drain into confluence and result in the formation of the portal vein. There is evidence to support the view that a partial partition of blood flow (streamline flow) exists in the portal vein^10^.

Remote metastases are reported in ~25–40% of breast cancers and out of these, 5% are classified as an advanced disease when diagnosed initially and lead to overall poor survival^11^. The third most common distant metastatic site after bone and lung is the liver which accounts for 7.3% of overall breast cancer metastases. The Association of Breast Cancer and Liver Metastases (BCLM) with poor prognosis and a median survival time of 2–3 years is reported^12^.

The likelihood of metastatic spread is influenced by the molecular subtype of breast cancer: there is a higher risk of metastatic relapse in women with ER/PR-negative tumours in the first 5 years as compared to women having ER/PR-positive tumours^13^. The association of lobar distribution of hepatic metastasis from colorectal adenocarcinoma with primary tumour localization has not been found significant^14^. However, the literature available on this topic is not sufficient to reach any conclusion. The present study aims to determine the frequency of segmental distribution of liver metastasis (LM) seeding from portal versus systemic routes of dissemination from primary CRC and breast carcinoma respectively.

## Material and methods

### Study design and setting

The study has a retrospective study design investigating outcomes specified by the data collected from a tertiary care teaching hospital in Pakistan. A retrospective chart review was performed that included all consecutive patients from 2018 to 2020.

### Study population

The study included adult patients who had been diagnosed with CRC and breast carcinoma and developed LM as the primary site of recurrence. The sample comprised individuals who had received treatment at the hospital within a specified time frame. The study population employed 587 patients comprising 179 male and 408 female patients of mean age 54.9±13.3.

### Data collection

A comprehensive data collection process was employed to gather relevant information. The medical records of eligible patients were carefully reviewed, and demographic data, clinical characteristics, and imaging findings were extracted. Specific variables of interest included the primary cancer type (CRC or breast carcinoma), the presence of liver metastasis, and the segmental distribution of metastatic lesions within the liver. Patients’ demographics and clinical information were collected on a predesigned performance.

### Imaging analysis

Computed tomography scans and other relevant imaging modalities were utilized to evaluate the segmental distribution of liver metastasis. Axial and multiplanar reformatted computed tomography images were reviewed by experienced radiologists to identify and localize the metastatic lesions within the liver. The location and involvement of specific liver segments were recorded for each patient. We excluded segment I from the calculation because of the dual blood supply.

### Statistical analysis

The collected data were analyzed using appropriate statistical methods in Statistical Package For The Social Sciences (SPSS) version 25. Descriptive statistics such as frequencies, percentages, means, and standard deviations were calculated to summarize the demographic and clinical characteristics of the study population. The frequency and distribution of liver metastasis among different liver segments were assessed. Statistical tests, including χ^2^ analysis, were employed to examine the differences in segmental distribution between portal and systemic routes of dissemination in CRC and breast carcinoma, respectively. Results with *P* value less than or equal to 0.05 were considered statistically significant.

### Study outcomes

The study revealed the incidence of metastasis in the right and left lobes of the liver due to CRC and breast cancer. Furthermore, metastatic involvement of the left lateral segment and medial segment due to breast and colon cancer was assessed.

### Ethical considerations

The anonymity and confidentiality of the patients involved were considered. The institutional review board approved the study protocol, ensuring the protection of patients’ rights and welfare.

## Results

**Figure 1:**
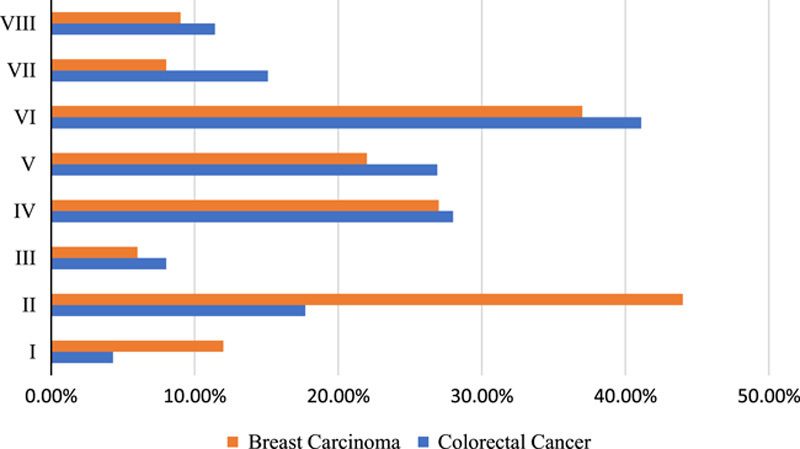
The graph depicts the percentage of hepatic metastasis in different segments of liver in correlation with breast carcinoma and colorectal cancer.

A total of 587 patients were included in the study. The study population comprised 179 (30.5%) male and 408 (69.5%) female patients. The mean age of the patients was 54.9 ± 13.3. There were 287 (48.9%) patients with CRC and 300 (51.1%) patients with breast carcinoma. The frequency and percentage of metastatic liver disease in Segment I–VIII are as follows: 69 (11.8%), 228 (38.8%), 222 (37.8%), 165 (28.15), 188 (32%), 277 (47.2%), 251 (42.8%), and 235 (40%), respectively (Figure 1).

Among all the patients, 204 (34.8%) CRC patients showed right lobe (V, VI, VII, VIII), and 83 (14.1%) showed left lobe metastasis. Among breast cancer patients 192 (32.7%) showed right lobe and 108(18.4%) showed left lobe metastasis (*P*=0.02). We also found 40 (6.8%) CRC and 55 (9.4%) breast carcinoma patients showed left lateral segment (II, III) involvement. Medial segment involvement (IV) was seen in 43(7.3%) patients with CRC and 53(9%) patients with breast carcinoma (*P*=0.03) as shown in Table 1.

**Table 1:** Differences between lobar distribution of colorectal carcinoma and breast cancer metastasis

Lobes (segments)		CRC *n* (%)	BC *n* (%)	Total *n* (%)
Right lobe ^*^ (V, VI, VII, VIII)		204 (34.8)	192 (32.7)	396 (67.5)
Left lobe (II, III, IV)		83 (14.1)	108 (18.4)	191 (32.5)
Left lobe	Lateral segment (II, III)^*^	40 (6.8)	55 (9.4)	95 (16.2)
	Medial segment (IV)	43 (7.3)	53 (9)	96 (16.4)
Total		287 (48.9)	300 (51.1)	587 (100)

BC, breast cancer; CRC, colorectal cancer.

* *P* value less than 0.05.

Segment I lesion were found in 36 (12%) of the breast cancer patients compared to 15 (4.3%) of the CRC patients. This had statistical significance (*P*=0.002). Segment II lesions were found in 132 (44%) breast cancer patients and 62 (17.7%) CRC patients. This was statistically significant (*P*=0.0001). Segment VII lesions were found in 24 (08%) of the breast cancer patients compared to 53 (15.1%) of the CRC patients.

This had statistical significance (*P*=0.04). There were no significant differences in lesions in the rest of the liver segments between the breast cancer and CRC groups. Table 2 depicts the distribution of hepatic lesions for all segments in the breast cancer and CRC groups.

**Table 2:** Differences in the segmental distribution of colorectal cancer and breast cancer metastasis to liver

Segment of the lesion	Colorectal cancer, *n* (%)	Breast cancer, *n* (%)	*P*
I	15 (4.3)	36 (12)	<0.05
II	62 (17.7)	132 (44)	<0.0001
IIII	28 (8)	18 (6)	>0.05
IV	98 (28)	81 (27)	>0.05
V	94 (26.9)	66 (22)	>0.05
VI	144 (41.1)	111 (37)	>0.05
VII	53 (15.1)	24 (8)	<0.05
VIII	40 (11.4)	27 (9)	>0.05
Total	534 (152.6)	495 (165)	>0.05

## Discussion

Clinical implications of knowledge encompassing the varying incidence of LM from primary CRC and breast carcinoma in different hepatic segments and the hepatic lobes may remarkably contribute in devising better surgical strategies, which is considered as first line of treatment in LM^8^. To the best of our knowledge, this is the first comprehensive study putting forward statistically significant outcomes regarding the patterns of hepatic involvement in LM from primary CRC and breast carcinoma suggesting greater involvement of right hepatic lobe as compared to the left hepatic lobe and more frequent involvement of hepatic segments I, II, and VII as compared to other hepatic segments. The incidence of LM is between 14 and 18% in patients with CRC while it is found to be increased by 10 and 25% in patients following resection of the primary tumour^7^. LM has considerable therapeutic implications and the understanding regarding its segmental distribution may help in contributing positively towards early diagnosis, better management strategies, and improved disease progression. Early diagnosis of carcinoma is associated with prolonged surveillance^5^ and subsequent better prognosis. This is the first study that evaluates the segmental distribution of LM from primary colorectal and breast carcinoma, while also taking into consideration the route of metastasis.

Several studies support varying involvement of morphological segmental patterns in LM as indicated by the results of our study. Kuo *et al.* ^15^ reported a greater incidence of multiple lesions with involvement of multiple segments, large metastatic deposits, and bilobar involvement with higher recurrence rates in centrally located metastasis as compared to peripheral involvement. Together with this, a recent study indicates that there is a statistically significant difference between the involvement of the right and left hepatic lobes with the right lobe being more affected than the left lobe (*P*<0.001)^16^. Rhu *et al*.^17^ also reported greater involvement of the right hepatic lobe in breast carcinoma patients as compared to the left hepatic lobe. A possible explanation for this may be greater anatomical angulation of the left portal vein branch^16^. As a heterogenous neoplasm, breast cancer can differentiate into cells with diverse growth rates and metastatic potential, tumour size, and histological grade, the knowledge of which can have significant therapeutic implications as are provided by a recent study which indicates a remarkable morphological discrepancy in primary and metastatic breast cancer putting forward a possibility for recognition of a better prognosis timely with appropriate management and treatment plans^18^.

Together with the anatomical discrepancy, the segmental distribution of LM may be influenced by the laterality of the primary tumour. CRC has been found to exhibit different incidence, pathogenesis, genetic involvement, molecular pathways, and prognosis depending upon the location of the tumour^19^. Lynch syndrome refers to the most common form of CRC and is found to have a better long-term survival as compared to sporadic colorectal carcinoma despite of statistically comparable recurrence-free survival^20^. Better long-term survival associated with Lynch Syndrome may indicate towards hereditary CRC less commonly involved with LM as compared to sporadic colorectal carcinoma. Another study supports that left-sided colorectal carcinoma is found to have a greater association with LM as compared to right-sided colon cancer^21^. In addition, Abdou *et al*.^22^ found that left-sided breast cancer has a more aggressive nature and worst outcomes as compared to ride-sided breast cancer and may have a greater tendency to develop hepatic metastasis. Breast carcinoma can present with varying histological and morphological characteristics in biopsy. invasive ductal carcinoma (IDC), invasive lobular carcinoma and mixed invasive ductal and lobular carcinoma (IDC-L) may metastasize to liver differently depending on their individual long-term prognoses and rate of recurrence. Metzger-Filhoand colleagues suggest that IDC-L have the best prognosis, especially in post-menopausal women^23^.

Another phenomenon that may explain the distinct lobar distribution of hepatic metastasis may be the streamlined flow of the portal vein. Venous drainage from the right and transverse colon drains into the superior mesenteric vein while venous drainage from the left colon and upper rectum drain into an inferior mesenteric vein. Subsequently, the portal vein is formed by the convergence of the superior mesenteric vein and splenic vein. This results in the mixing of blood in the port vein from inferior and superior mesenteric veins constituting streamlining of blood flow suggesting disproportionate blood supply to the right and left hepatic lobes giving rise to a distinct lobar distribution of hepatic metastasis through the portal vein. Rhu *et al*.^17^ found that there is a significant difference in the lobar distribution of hepatic metastases from left and right-sided colorectal carcinoma and also put forward that the survival of left hepatic lobe metastasis is poorer than left-sided. Different surgical approaches and treatment regimens such as preoperative portal vein embolization and systemic chemotherapy devised according to the distinct source of seeding in consideration for specific segments in LM may also be linked with an increased rate of recurrence in the same segments, putting forward a possibility of greater involvement of certain segments than others as indicated by the results of our study. However, surgical strategies for the resection of hepatic metastasis are defined according to intraparenchymal and peripheral locations of the metastatic deposits^24^. Surgical resection despite being the only resort for long-term survival often presents with recurrence. Multimodal management inculcating open laparoscopic surgeries and ablation therapies such as radiofrequency ablation and (neo)adjuvant chemotherapy may help in better long-term prognosis with decreased recurrence rates^25^. In addition, Ishiwaza *et al*.^26^ evaluated surgical strategies to complete total laparoscopic segmentectomy for all hepatic segments (I–VIII) and found affirmation of the feasibility of its approach in minimizing the loss of functional liver volume without reducing its curability. The knowledge of hepatic segments I, II, and VII being most frequently involved, as put forward by our study may have significant clinical implications in extension of existing total laparascopic segmentectomy strategy with better plans of compound segmentectomies of hepatic segments which present the greatest risk of being affected by recurrence. In terms of management, early introduction of right hepatic lobe screening in the screening protocol of CRC and breast carcinoma may prove effective in detecting LM timely and devising better therapeutic plans accordingly.

The work has been reported in line with the STROCSS 2021 criteria^27^.

## Limitations

Firstly, the retrospective study design contains inherent bias. Secondly, the results of the study cannot be generalized to the rest of the population. Lastly, this study could not establish causality between the exposure (portal versus systemic route of metastasis) and the outcome (the tendency of segmental distribution of hepatic metastasis).

## Conclusion

The distribution of hepatic metastasis in segments I, II, and IV shows statistically significant differences. Lesions in segments I and II were more frequent in the breast cancer group, while segment VII lesions were more frequent in the CRC group. The distribution of hepatic lesions in the remaining liver segments did not differ significantly between the breast cancer and CRC groups.

## Ethical approval

Protocol number: 2021-6533-19437.

Ethical approval for this study (Ethical Committee AKUH) was provided by the Ethical Committee of Aga Khan University Hospital, Karachi, Pakistan on 10 October 2021.

## Consent

Written informed consent was obtained from the patient for publication and any accompanying images. A copy of the written consent is available for review by the Editor-in-Chief of this journal on request.

## Sources of funding

None.

## Author contribution

D.K.: idea conceptualization, study design. A.K.K.: idea conceptualization, study design, writing original draft. S.D.K.: data collection, data analysis. M.A.: data collection, writing original draft. A.A.: data collection, data analysis, writing. M.W.: writing original draft, reviewing, proof reading. U.K.: editing, proof reading, project management. F.K., A.P., A.K.: data collection, project management.

## Conflicts of interest disclosure

The authors report no actual or potential conflicts of interest.

## Research registration unique identifying number (UIN)

1. UIN number: Researchregistry9070.
2. Registry used: research registry.
3. Hyperlink: https://www.researchregistry.com/browse-theregistry#home/?view_2_search=researchregistry9070&view_2_page=1.

## Guarantor

Dawar Khan.

## Provenance and peer review

Not commissioned, externally peer-reviewed

## Data statement

No data are associated with this submission.
